# Effectiveness of the attachment position in molar intrusion with clear aligners: a finite element study

**DOI:** 10.1186/s12903-022-02472-z

**Published:** 2022-10-27

**Authors:** Dian Fan, Hao Liu, Chang-Yong Yuan, Shi-Yu Wang, Peng-Lai Wang

**Affiliations:** 1 School of Stomatology, Xuzhou Medical University, Xuzhou, China; 2 Department of Orthodontics, Affiliated Stomatological Hospital of Xuzhou Medical University, Xuzhou, China; 3 Department of Implantology, Affiliated Stomatological Hospital of Xuzhou Medical University, Xuzhou, China

**Keywords:** Clear aligners, Finite element analysis, Molar intrusion, Attachment

## Abstract

**Objective:**

To evaluate the biomechanical effects of different attachments’ position for maxillary molar intrusion with clear aligner treatment by finite element analysis.

**Methods:**

Cone-beam computed tomography images of a patient with supra-eruption of the maxillary second molars were selected to construct three-dimensional models of the maxilla, periodontal ligaments, dentition, and clear aligner. The models were divided into four groups depending on the attachment location on the first molar: (1) no attachment (NA), (2) buccal attachment (BA), (3) palatal attachment (PA), and (4) bucco-palatal attachment (BPA). After applying an intrusion of 0.2 mm on the second molar, displacements and stress distributions of the teeth, aligner, and periodontal ligament were analyzed with the finite element software.

**Results:**

All groups displayed equivalent movement patterns of aligners. The NA and BA groups showed buccal tipping of the second molar, while the PA group showed palatal tipping. The BPA group had the highest intruding value and the lowest buccal/palatal tipping value. All groups showed mesial tipping of the second molar. Stress distribution in the periodontal ligament strongly correlated with the attachment position. The BPA group showed the best stress distribution.

**Conclusion:**

Combined BA and PA could effectively prevent buccal and palatal tipping and showed the best efficiency in intruding the second molar. The second molar showed an unavoidable tendency to tip mesially, regardless of the attachment position.

## Introduction

In the past two decades, the clear aligner has been increasingly used owing to its esthetic and transparent features [1]. Aligners are effective in teeth intrusion, as they cover the entire dentition, exhibiting the “block effect” on the molars [2, 3]. In a retrospective study, clear aligners showed excellent clinical vertical control of the molars [4]. The intruding posterior teeth with clear aligners were also a correction strategy for open bite patients [5–7]. However, the biomechanical information in the molar intrusion movement remains unknown.

Unlike fixed appliances, the clear aligner is composed of thermoplastic materials and attachments, which provide a consistent and gentle force [8]. For esthetic purposes, attachments are mainly used as retention aids made of resin and bonded to the target teeth surface [9]. They can change the direction of orthodontic forces applied to the teeth to guide them toward the target position. Hong et al. found that adding attachments could aid in achieving orthodontic intrusion movements [10]. The attachment position is essential for intrusion. However, no studies have evaluated the effects of different attachment positions on the intrusion movement of the maxillary molars [11].

A finite element analysis (FEA) can be used to divide the dentition into a finite size and number of units and study the properties of each unit to obtain the overall properties. Several studies have extended the application of FEA in the evaluation of orthodontic forces [12, 13]. Currently, it is the best method to analyze orthodontic forces [14]. Therefore, it could be used to simulate the clinical environment of clear aligners [15].

The aim of this study was to evaluate the effects of different attachment positions on the intruding maxillary molars and analyze the displacements and stress distributions of the teeth, aligner, and periodontal ligament (PDL) and establish an optimal attachment protocol to guide clinical practice.

## Materials and methods

### Data collection and model establishment

A 25-year-old woman with a supra-erupted maxillary second molar (class I, excluding other influencing experimental diagnoses) was enrolled for the study. Cone-beam computed tomography (CBCT) data were obtained, and a total of 503 images were exported to Mimics Research version 17.0 (Materialise, Leuven, Belgium) to reconstruct the maxilla along with the teeth. The study protocol was approved by the Ethics Committee of the Affiliated Stomatological Hospital of Xuzhou Medical University (2021-012). The subject provided written informed consent. All methods were performed in accordance with the relevant guidelines and regulations.

**Fig. 1:**
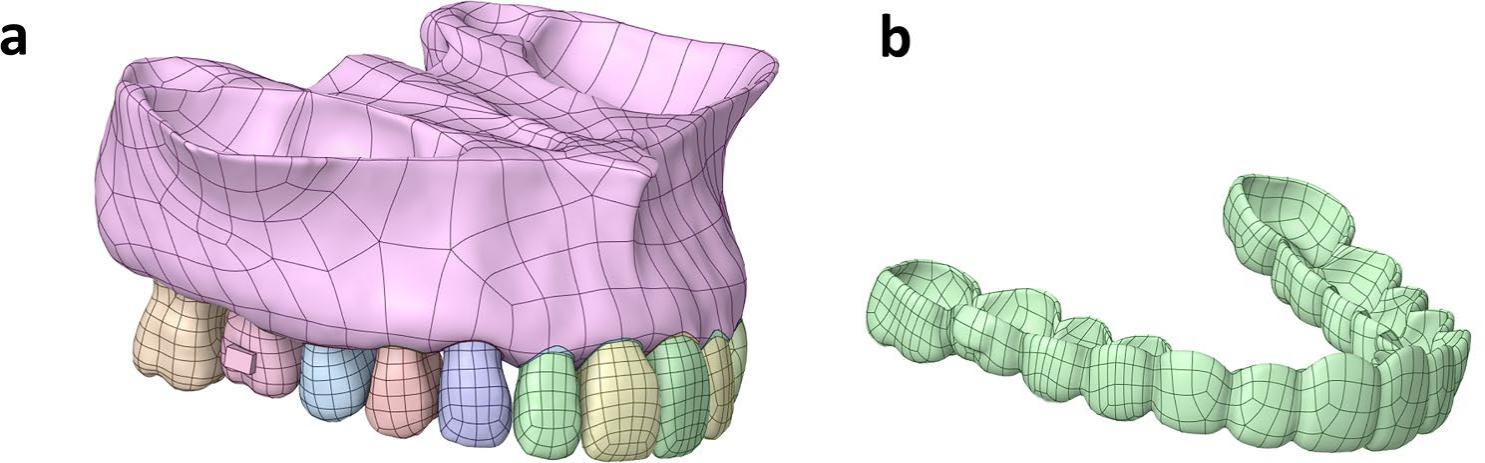
Finite models. (a) Models of the maxilla, periodontal ligament, dentition, and attachment. (b) Model of the clear aligner.

First, all extracted teeth models were externally offset in Geomagic Wrap 2017 (Geomagic, North Carolina, USA) to obtain the PDL models, with a thickness set to 0.25 mm [16]. Second, the crown was offset externally by 0.75 mm to model the aligners [17, 18]. Alveolar socket models and PDL of the maxilla were created using the Boolean operation in SolidWorks 2021 (Dassault Systèmes, Massachusetts, USA). Third, a horizontal rectangular attachment, with dimensions of 3 mm × 2 mm × 1 mm, was built using SolidWorks and positioned at the center of the clinical crown [9, 19] (Fig. 1).

### Material properties and operation condition

**Table 1:** Material properties

	Young’s modulus (MPa)	Poisson’s ratio
Teeth	1.96*10^4^	0.30
Periodontium	0.67	0.45
Alveolar bone	1.37*10^4^	0.30
Attachment	1.25*10^4^	0.36
Aligner	528	0.36

**Table 2:** Number of nodes and elements in each part

	Nodes	Elements
Teeth	74,359	41,342
Periodontium	133,498	69,255
Alveolar bone	59,538	35,617
Attachment	298	123

In material properties, the individual parts were linearly elastic, isotropic, and homogeneous, as described in previous studies [19, 20] (Table 1). All the models were divided into tetrahedral meshes. Table 2 shows the number of nodes and elements generated during the discretization process.

Fixed support was applied to the upper surface of the maxilla with 0 degrees of freedom in six directions. The contact conditions of PDL with the teeth and the alveolar sockets were set to bond. Bonded contact was established between the attachment and the teeth. Simultaneously, friction contacts were applied for clear aligners with the teeth and the attachment, with a Coulomb friction coefficient of 0.2 [21].

**Fig. 2:**
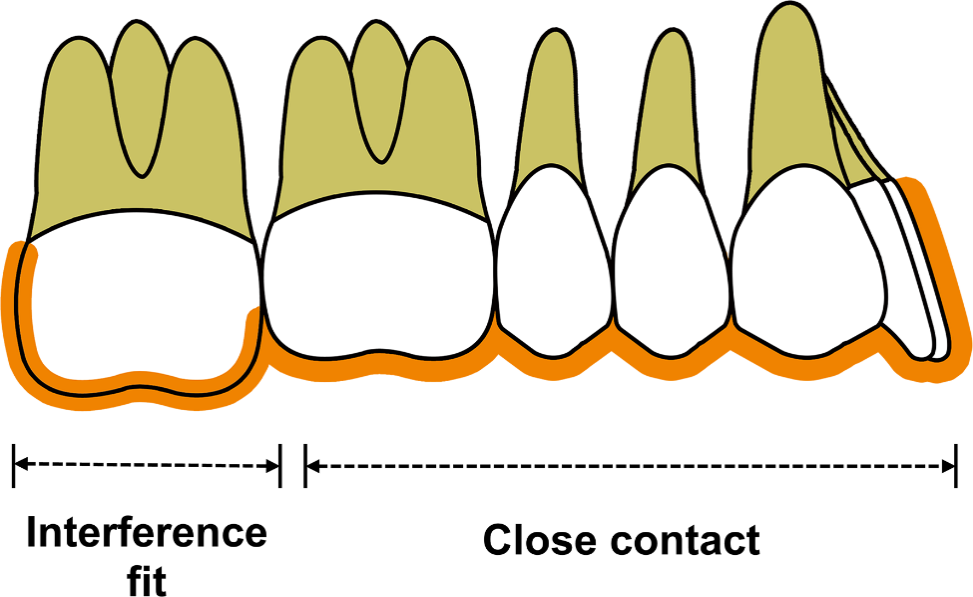
Loading method. Orange represents the clear aligner model. The model intrusion section contains an interference, with the remaining portion set to close contact.

A new load approach was developed to simulate the actual clinical environment. First, finite element models of the aligner were established with an intrusion of 0.2 mm in the maxillary second molar, creating an interference between the aligner and the molar surface. Subsequently, we set close contact between the anchorage teeth and the corresponding part of the aligner, and set the intrusion part of the molar with an interference fit. The amount of interference was equal to a preset intrusion amount of 0.2 mm for the aligner (Fig. 2).

### Coordinate system and model grouping

The initial coordinate system was established from CBCT data. The X-axis represented the coronal direction, with the positive direction representing the palatal side. The Y-axis represented the sagittal direction, with the positive direction representing the distal side. The Z-axis represented the vertical direction, with the positive direction representing the gingival side.

**Fig. 3:**
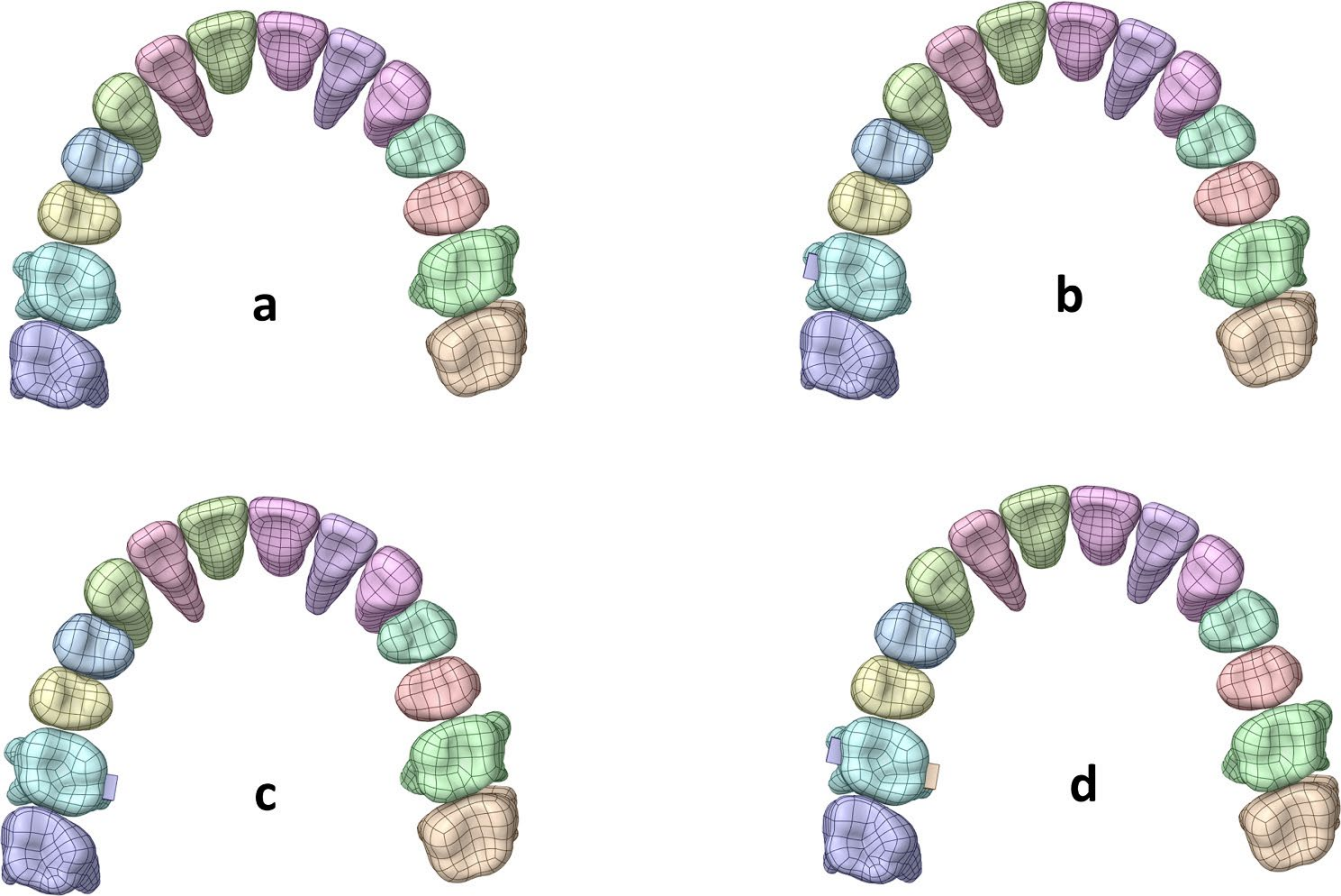
Four model groups. (a) No attachment. (b) Buccal attachment. (c) Palatal attachment. (d) Bucco-palatal attachment.

Four model groups were divided based on the attachment position. After applying a 0.2 mm intrusion on the second molar [22], the movement pattern of the aligners, the stress distribution of PDL, and displacement tendencies of the intrusion and the anchorage teeth were analyzed (Fig. 3).

Group A: No attachment (NA).

Group B: First molar buccal attachment (BA).

Group C: First molar palatal attachment (PA).

Group D: First molar bucco-palatal attachment (BPA).

## Results

### Movement pattern of clear aligners

**Table 3:** Aligner deformation and location

Group	Maximum deformation(mm)	Maximum deformation location	Minimum deformation(mm)	Minimum deformation location
NA	0.357	Distal surface of the right second molar	0.000	Distal surface of the left second molar
BA	0329	Distal surface of the right second molar	0.000	Distal surface of the left second molar
PA	0.330	Distal surface of the right second molar	0.000	Distal surface of the left second molar
BPA	0.315	Distal surface of the right second molar	0.000	Distal surface of the left second molar

**Fig. 4:**
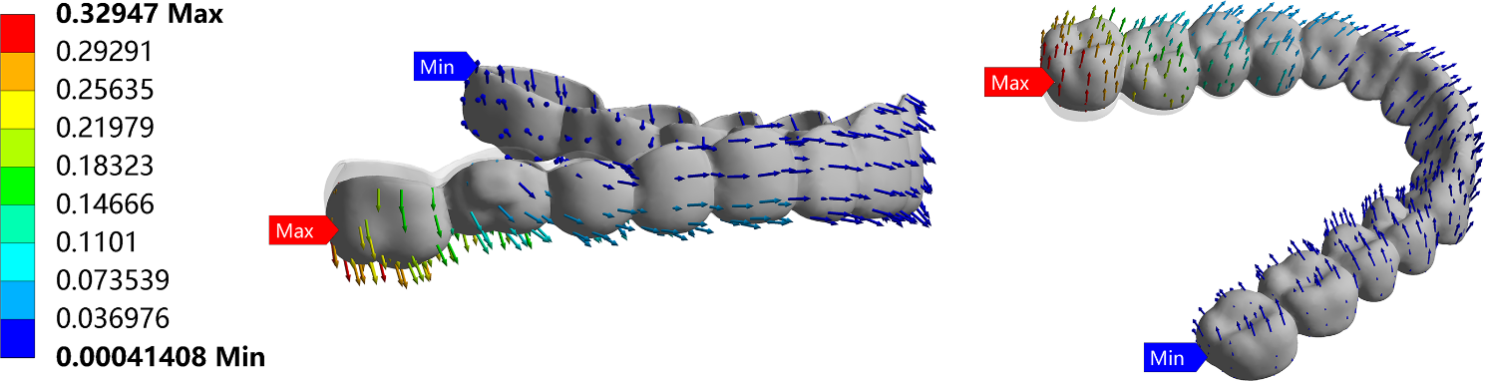
Movement pattern of clear aligners. The undeformed aligner is translucent.

Aligners moved toward the occlusal surface of the posterior teeth, particularly in the second molar region, and were displaced forward in the anterior teeth. Deformation of the entire aligner gradually decreased from back to front. All groups showed the same movement pattern of aligners (Fig. 4).

The maximum deformation on the aligner occurred on the distal surface of the right second molar. In contrast, the minimum deformation occurred on the distal surface of the left second molar. The maximum deformation appeared in the NA group (0.357 mm), followed by the PA (0.330 mm), BA (0.329 mm), and BPA (0.315 mm) groups (Table 3).

### Displacement trend of the intrusion tooth

**Table 4:** Three-dimensional displacements of the upper second molar (Unit:µm)

Displacement			Groups			
**orientation**	**Measurement Point**		**NA**	**BA**	**PA**	**BPA**
X	MB		-15.33	-39.26	16.64	-5.55
	ML		-6.52	-31.20	27.30	4.57
	DB		-2.76	-24.50	27.32	7.44
	DL		3.77	-16.94	32.99	14.23
Y	MB		-23.29	-34.67	-30.12	-45.44
	ML		-42.06	-57.24	-48.12	-67.69
	DB		-20.68	-31.48	-27.63	-42.31
	DL		-33.65	-46.30	-39.12	-55.80
Z	MB		17.95	42.25	24.90	47.26
	ML		5.53	11.72	27.19	30.65
	DB		3.47	18.44	3.18	21.88
	DL		-5.64	-1.59	6.58	6.92

Note. X-axis: (+) for palatal and (-) for buccal; Y-axis: (+) for distal and (-) for mesial; Z-axis: (+) for intrusion and (-) for extrusion

**Fig. 5:**
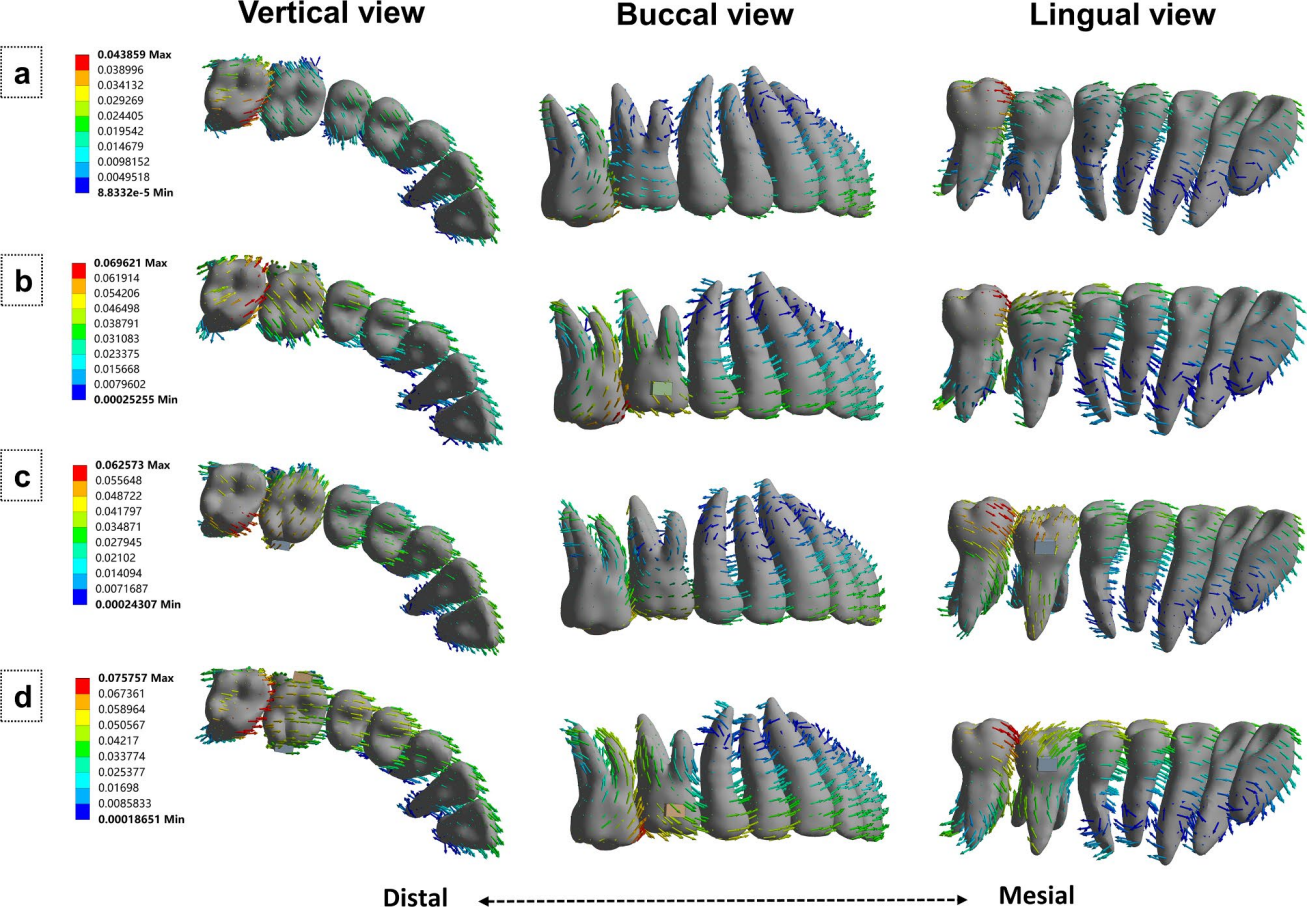
Displacement trend of the dentition. (a) No attachment; (b) Buccal attachment; (c) Palatal attachment; (d) Bucco-palatal attachment.

Four points of the maxillary second molar, i.e., the mesiobuccal (MB), mesiolingual (ML), distobuccal (DB), and distolingual (DL) cusps, were selected to measure the three-dimensional displacement. Table 4; and Fig. 5 show the results.

On the X-axis, all points in the BPA group showed the lowest displacement, with significant differences compared to the BA and PA groups. All points in the BA group showed relatively more buccal displacements compared to the NA group, while all points in the PA group showed palatal movements. On the Y-axis, all points in all groups shifted mesially. The BPA group showed the highest displacement, while the NA group showed the lowest. Displacements in the BA and PA groups did not differ. On the Z-axis, all points in the BPA group showed the highest displacement. The sum of the mesial measurement point (MB + ML) displacements was greater than that of the distal measurement point (DB + DL) displacements in all groups.

### Displacement trend of the anchorage teeth

Displacement trends of the first molar differed among the four groups (Fig. 5). In the NA group, the crown shifted distobuccally, while the roots shifted mesiolingually, resulting in a clockwise motion of the first molar. In the BA group, the crown shifted mesiolingually, while the roots shifted occlusally, producing mesiolingual tipping trends. In the PA group, the crown shifted buccally with slight mesial tipping, while the roots shifted distally. In the BPA group, the first molar achieved bodily mesial tipping, with rare shifts buccally or lingually.

Moreover, the anterior and premolar displacement trends in the non-control groups were almost the same. For the premolars, the crowns tipped mesially, while the roots shifted distally, showing mesial tipping. In the control group, the teeth moved mesially with lingual inclination. For the anterior teeth, the groups showed no differences. The crowns of all anterior teeth shifted mesially, exhibiting labial tipping trends.

### Stress distribution in PDL

**Fig. 6:**
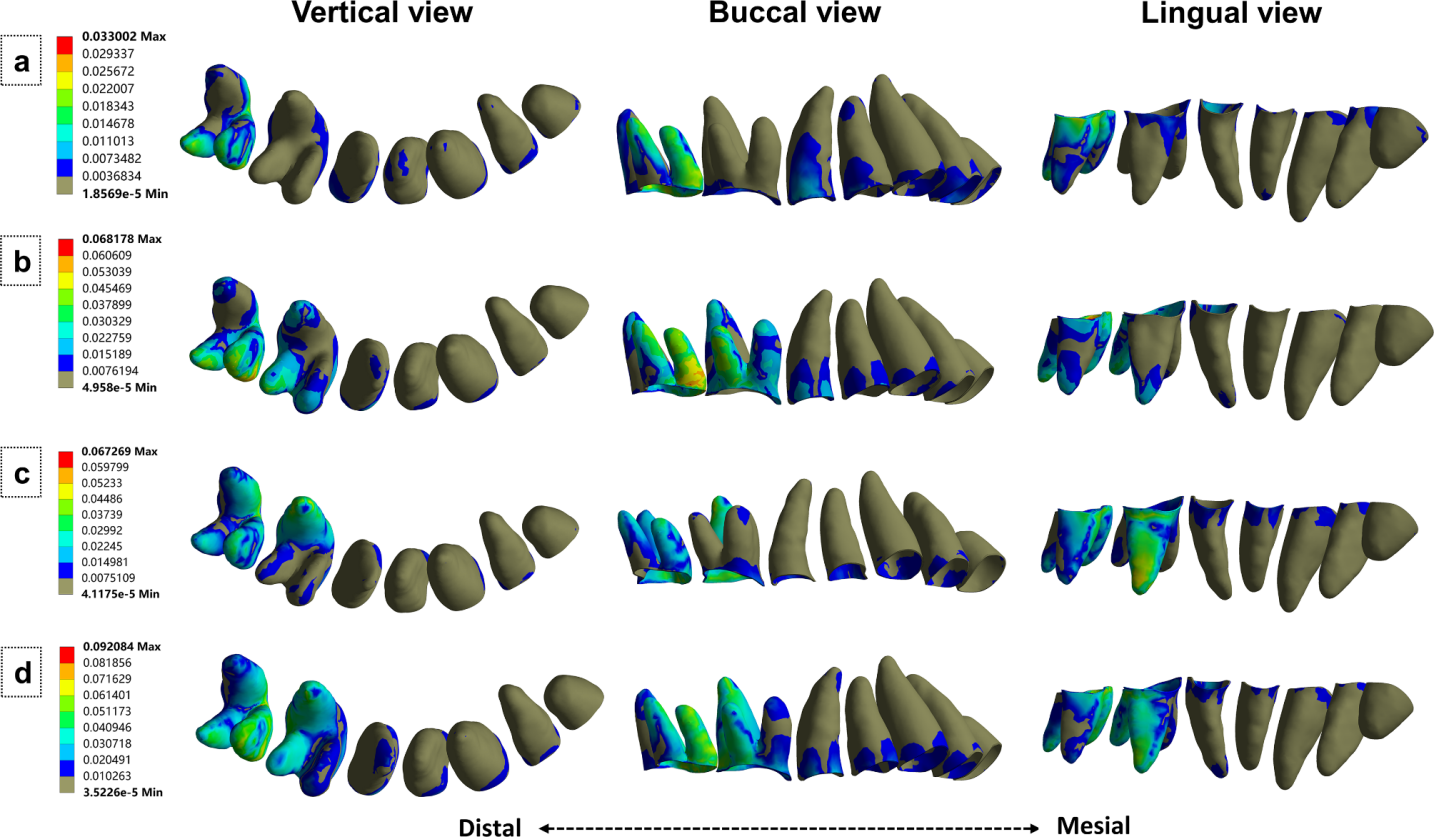
Equivalent stress distribution in periodontal ligament. (a) No attachment. (b) Buccal attachment. (c) Palatal attachment. (d) Bucco-palatal attachment.

The stress distribution also differed among the four groups (Fig. 6). In the NA group, the stress was distributed on the buccal roots, particularly the mesiobuccal root of the second molar. In the BA group, the stress was distributed on the buccal roots and palatal root apices of the first and second molars, with minor stress on the buccal surface of the anterior teeth and the premolar neck. In the PA group, the stress was mainly distributed on the second molar and the palatal surfaces of the roots of the other teeth, resulting in a palatal stress distribution of the anchorage teeth. In contrast, in the BPA group, the stress was well-distributed and more uniform over the molar roots, while the distribution pattern was similar to that of the BA group in the anterior teeth and the premolars.

**Fig. 7:**
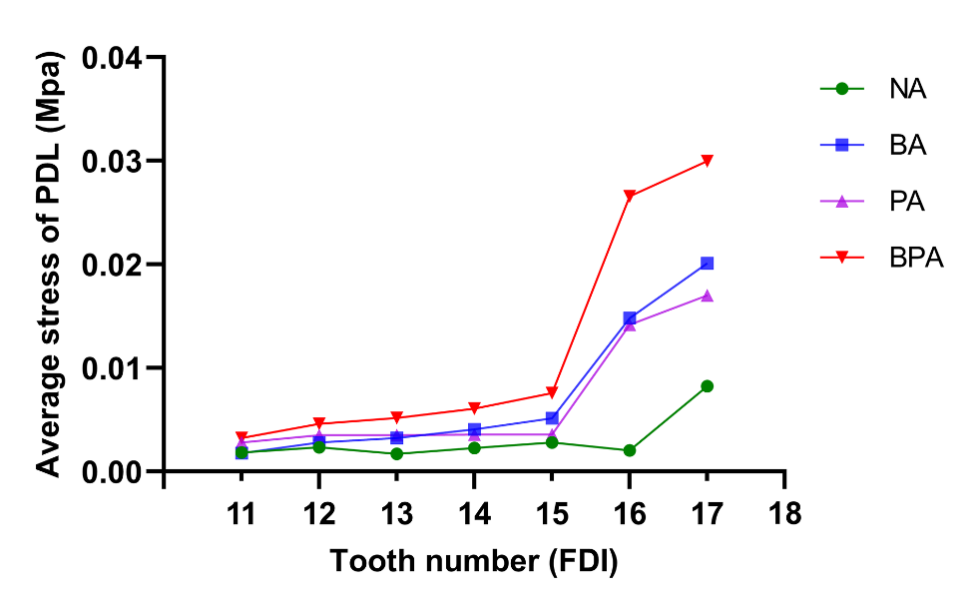
Average equivalent stress in the periodontal ligament.

Figure 7 shows the average stress on PDL. The BPA group showed the highest PDL pressure. However, the stress difference only became apparent in the molars (#16 and #17). The difference between the BA and PA groups was insignificant. The three non-control groups showed a gradual stress increase from the anterior teeth and the premolars (#11 to #15) and a sharp increase in the molars (#16 and #17).

## Discussion

This was the first study to examine the intruding molars’ biomechanical behaviors with FEA and successfully obtain biomechanical information for different attachment positions. Considering that the intruded tooth was located in the most distal portion of the dentition, we chose the horizontal rectangular attachment with higher intrusion efficiency and set it on the adjacent tooth to obtain the best effect [23]. The effectiveness of rectangular attachments in tooth intrusion movement has been demonstrated in prior studies [24, 25]. Moreover, only the intrusion side of the dentition was shown because the data on the non-intrusion side were insignificant and not the primary point of our study.

The movement pattern of the clear aligners directly reflects the way the load is applied. Different ways of applying force can affect the overall results[19, 20]. In clinical practice, when clear aligners are used to intrude the molars, the amount of intrusion is pre-programmed before the aligner is placed into the target tooth. The force applied to the teeth depends on the aligner material and the amount of intrusion. This is similar to how interference fit generates force[26]. Therefore, we set up an interference fit between the intrusion tooth and the aligner. The results validated the reliability of the method. We found the same aligner movement pattern in all four groups, reflecting the clinical scenario. Particularly for the second molar, aligners moved away from the tooth surface, consistent with the FEA results in Rossini et al.’s study [27].

The ability of a clear aligner to intrude a tooth is determined by the force exerted on the tooth [28]. Aligner deformation is a major factor affecting clear aligner therapy results [29]. In our study, the difference between BA (0.329 mm) and PA (0.330 mm) in aligner deformation was insignificant, indicating a close force generated by the aligners. However, compared to the NA group, deformation decreased with increase in the number of attachments. The increase in attachment may lead to a better retention performance for aligners (smaller deformation), which generate more intrusion force on the teeth. Consequently, BPA appears to be better to intrude molars.

Vertical control of the teeth has been a challenging issue in clear aligner therapy, especially the “bowing effect” that may exist when intruding the premolars and the anterior teeth [16, 30]. In the sagittal direction (Y-axis), our study showed that regardless of the attachment position, an anticlockwise moment was generated during intrusion movement, causing the second molar to tilt mesially. A similar phenomenon was found in a micro-sensor study by Zhu et al. [31]. The “bowing effect” seems to be inevitable. However, this phenomenon did not present in several clinical studies because of blockage of the adjacent teeth and coverage of the aligners [5, 32, 33]. Further studies are warranted to confirm these findings.

In the horizontal direction (X-axis), applying BA led to whole buccal tipping of the tooth, whereas applying PA led to whole palatal tipping of the tooth. The BPA group also showed slight buccal tipping. Considering that the maxillary posterior teeth have a natural buccal torque [34], the layout of BPA may have the best performance in reducing uncontrolled buccal or palatal tipping.

In the vertical direction (Z-axis), the notable difference between the control and non-control groups indicated the necessity of attachments for the intruding molar. The BPA group exhibited the best intrusion efficiency. Bowman et al. found that the mesial aspects of the molars tended to intrude, while the crowns tipped forward when intruding the molars with clear aligners [35]. These findings were consistent with our study, which showed that the intrusion amount at the mesial measurement points was over twice that of the distal measurement points. From a biomechanical view, the MB and ML cusps of the maxillary second molars had a larger volume than the corresponding distal cusps, and they are closer to the anchorage, bearing higher pressure. This results in a greater moment in the mesial cusps of the crown than in the distal ones, letting the crown move anticlockwise. Therefore, orthodontists should be cautious regarding the attachment position, avoiding unbalanced moments in the teeth.

Stability of anchorage is the premise for achieving expected effects with clear aligners. During each treatment step of clear aligners, shape mismatches between the aligner and the target tooth surface would produce a force system directly transmitted to the teeth, producing tooth movement [36]. The increase in this mismatch may lead to undesired movements near the anchorage unit [29]. In our study, only the BPA group displayed the same displacement trends as the aligners. The BA and PA groups could result in an unfavorable displacement of the anchorage teeth, probably because of the fact that the double attachments of the BPA group increased the retention force of the aligner and reduced displacement of the anchorage teeth.

The stress in PDL is also a significant problem that cannot be ignored. It is produced by the aligner and transmitted through the tooth tissue to the periodontium[37]. In our study, the BPA group displayed better stress distribution. Neither the buccal nor palatal groups showed uniform stress distribution. Stress distribution was associated with the position of the attachment. In addition, the average PDL stress was higher in the BPA group. Several FEA studies have revealed a correlation between the increasing stress in the periodontium and root resorption[38, 39]. A higher stress may increase the possibility of periodontal tissue damage or even root resorption. Further research is required to elucidate the long-term clinical efficacy of BPA.

However, this study has several limitations. First, attachments were concentrated on the first molar in small numbers. The effects of the position and the shape of other attachments remain to be evaluated. Second, we simulated the single molar situation, whereas multiple and non-terminal cases remain uninvestigated. Third, the conclusion drawn from the simulation experiment should be confirmed with clinical cases.

### Conclusions

- The position of the attachment can affect the molar intrusion effect, and the attachment is essential for clear aligners to intrude the molars.
- Combined BA and PA have the optimal intrusion effect and reduce uncontrolled buccal or palatal tipping.
- The second molar shows an unavoidable tendency to tip mesially, regardless of the attachment position, and the result should be validated with more clinical cases.

## Data Availability

The datasets used and/or analyzed during the current study are available from the corresponding author on reasonable request.

## Abbreviations

FE: finite element
FEA: finite element analysis
PDL: Periodontal Ligament.
